# Lung inflammation following a single exposure to swine barn air

**DOI:** 10.1186/1745-6673-2-18

**Published:** 2007-12-18

**Authors:** Lakshman Nihal Angunna Gamage, Chandrashekhar Charavaryamath, Trisha Lee Swift, Baljit Singh

**Affiliations:** 1 Department of Veterinary Biomedical Sciences, University of Saskatchewan, Saskatoon, Canada; 2 University of Alberta, Edmonton, AB, Canada; 3 Immunology Research Group, University of Saskatchewan, Saskatoon, Canada

## Abstract

**Background:**

Exposure to swine barn air is an occupational hazard. Barn workers following an eight-hour work shift develop many signs of lung dysfunction including lung inflammation. However, the in situ cellular and molecular mechanisms responsible for lung dysfunction induced following exposure to the barn air remain largely unknown. Specifically, the recruitment and role of pulmonary intravascular monocytes/macrophages (PIMMs), which increase host susceptibility for acute lung inflammation, remain unknown in barn air induced lung inflammation. We hypothesized that barn exposure induces recruitment of PIMMs and increases susceptibility for acute lung inflammation with a secondary challenge.

**Methods:**

Sprague-Dawley rats were exposed either to the barn or ambient air for eight hours and were euthanized at various time intervals to collect blood, broncho-alveolar lavage fluid (BALF) and lung tissue. Subsequently, following an eight hour barn or ambient air exposure, rats were challenged either with *Escherichia coli (E. coli) *lipopolysaccharide (LPS) or saline and euthanized 6 hours post-LPS or saline treatment. We used ANOVA (P < 0.05 means significant) to compare group differences.

**Results:**

An eight-hour exposure to barn air induced acute lung inflammation with recruitment of granulocytes and PIMMs. Granulocyte and PIMM numbers peaked at one and 48 hour post-exposure, respectively.

Secondary challenge with *E. coli *LPS at 48 hour following barn exposure resulted in intense lung inflammation, greater numbers of granulocytes, increased number of cells positive for TNF-α and decreased amounts of TGF-β2 in lung tissues. We also localized TNF-α, IL-1β and TGF-β2 in PIMMs.

**Conclusion:**

A single exposure to barn air induces lung inflammation with recruitment of PIMMs and granulocytes. Recruited PIMMs may be linked to more robust lung inflammation in barn-exposed rats exposed to LPS. These data may have implications of workers exposed to the barn air who may encounter secondary microbial challenge.

## Background

Swine production is a major agricultural business in North America [1-3]. These days thousands of pigs are raised in large confinement buildings compared to small-scale family-managed operations in the past. Modern pig production operations increasingly employ full-time workers who might spend up to 8 hours/day inside the barn compared to 1–2 hours of work on the small farm in the past [4-6]. Although the environment inside these confinement buildings appears to be clean, it contains high levels of endotoxins, dust, bacterial DNA and gases such as ammonia and hydrogen sulfide [7-9].

There are evidences for reduced forced expiratory volume in one second (FEV1), wheeze and increased airway hyperresponsiveness (AHR) following a single exposure of naïve workers/volunteers to the barn air [10-12]. Sputum and bronchoalveolar lavage (BAL) obtained after a three hour exposure to the barn air showed increased levels of interleukin (IL)-8, IL-6, tumor necrosis factor-α (TNF-α), fibronectin and albumin [13-15]. There was also a 75-fold increase in the neutrophils and 2–3 fold increase in mononuclear cells in BAL from human naive volunteers exposed to the barn air for 3–5 hours while leukocyte counts increased in peripheral blood within 6 hours of an exposure [15]. Recent in vitro data show that swine barn dust stimulates secretion of IL-1β and TNF-α from alveolar macrophages and epithelial cells and expression of adhesion molecules on epithelial cells [16]. Recently, we have used a rat model to report that a single exposure to the barn air induces lung inflammation and AHR [17]. However, it remains unknown if an exposure to the barn air alters lung susceptibility to secondary challenges with microbes or their components such as lipopolysaccharide (LPS).

Lung inflammation is characterized by recruitment of neutrophils and monocytes into the alveolar septa and air spaces [18]. Monocyte recruitment into alveolar spaces in inflamed lungs is important for the removal of inflammatory debris and repair of tissue damage. However, prior to the entry of monocytes into alveolar spaces, they undergo physical interaction with the endothelium of lung capillaries and consequently may be retained in the capillaries. This is supported by our previous observations on the transient recruitment of pulmonary intravascular monocytes/macrophages (PIMMs) in lung inflammation in a rat model of sepsis [19,20]. We have also shown that PIMMs, similar to resident pulmonary intravascular macrophages in some domestic animal species, may alter the lung responses to a subsequent challenge [21-23,20]. Currently, there are no data on the recruitment and biology of PIMMs in animals exposed to the barn air.

Considering the putative critical roles of PIMMs, we undertook a series of in vivo studies to characterize PIMM recruitment and their functions in lung inflammation induced following exposure to the barn air. The data from these experiments demonstrate recruitment of neutrophils and PIMMs following a single exposure to the barn air and show a linkage between recruited PIMMs and increased lung inflammation following a secondary challenge with *E. coli *LPS.

## Methods

### Animal exposures

The University of Saskatchewan Committee on Animal Care Assurance approved protocols for the use of experimental animals in this study. Sprague-Dawley rats were kept in a swine barn for 8 hours. The cages were hung from the barn ceiling at a height of 5 feet from the floor. The rats were taken out of the barn at the end of the exposure and maintained in ambient air until euthanasia.

### Experiment 1: Effect of exposure to the swine barn air

Rats (N = 25) exposed to the barn air were euthanized 1 hour, 24, 48, 72 and 120 hours post-exposure (n = 5 for each time point). Control rats (N = 5) were kept in clean air prior to euthanasia.

### Experiment 2: Effect of secondary challenge with *E. coli *LPS on barn air exposed rats

Based on the data from Experiment 1, rats were administered either *E. coli *LPS (Sigma Chemical Co. St. Louis, MO, 1.5 μg/g body weight intravenous; n = 5) or saline (0.5 mL intravenous; n = 5) at 48 hours after an 8 hour exposure to the barn air. Unexposed rats were treated with *E. coli *LPS (n = 5) or saline (n = 5).

### Blood and broncho-alveolar lavage fluid (BALF) collection and analyses

At the end of the designated time points in Experiment 1 and 2, rats were euthanized (1 mg xylazine and 10 mg ketamine/100 g) for collection of blood, BALF and lung samples. Blood was collected by cardiac puncture for differential and total leukocyte counts. BALF was collected by lavaging the whole lung with three mL of ice cold Hanks Balanced Salt Solution (Sigma Chemicals Co., St. Louis, MO). The BALF was stored on ice until processing for total and differential leukocyte counts.

### Lung tissue processing

Left lung was snap frozen in liquid nitrogen, stored at -80°C and was later used in ELISA.

Right lobes of the lung were fixed in situ by instilling 4% paraformaldehyde in phosphate buffered saline (0.0016 M NaH_2_PO_4_, 0.008 M Na_2_HPO_4 _and 0.15 M NaCl), pH 7.2 for 30 minutes followed by immersion in the same fixative for 16 hours at 4°C. Three pieces collected from right lung were dehydrated, and embedded in paraffin. Five to seven μm thick sections were prepared and placed on glass slides coated with Vectabond (Vector Labs) and incubated at 55°C for 30 minutes to increase adherence of sections. Lung sections were stained with hematoxylin and eosin for histopathological assessment.

### Immuno-histochemistry and cell counts

Lung sections were processed for immunohistochemistry as described previously [24]. Briefly, sections were deparaffinized, dehydrated, treated with hydrogen peroxide (5% in methanol) to neutralize endogenous tissue peroxidase and exposed to pepsin (2 mg/ml 0.01 N HCl) to unmask the antigens. The sections were incubated with primary antibodies against monocytes/macrophages (ED-1, 1:75; Serotec, USA), granulocytes (HIS-48 1:50; BD Bioscience Canada), IL-1β (1:100; Santa Cruz Biotechnology, Inc., USA), TNF-α and TGF-β2 (1:75; R&D Systems, Inc., USA) for 60 minutes followed by incubation with appropriate secondary antibodies conjugated with horseradish peroxidase (1:100–1:250) for 30 minutes. Controls included staining without primary antibody or anti-von Willebrand Factor (vWF) antibody, which recognizes vascular endothelium, or with isotype-matched immunoglobulins. These sections were counter-stained with methyl green and immunohisotchemically positive cells in the lung septum were counted in 10 high power fields (400×; 0.096 mm^2 ^per field) under oil-immersion objective by a person blinded to the design of the experiment.

### Immuno-electron microscopy

Lungs samples were prepared for immuno-electron microscopy as described previously [25]. Briefly, tissues fixed in 0.1% glutaraldehyde and 2.0% paraformaldehyde in 0.1 M sodium cacodylate buffer for 3 hours at 4°C, dehydrated and infiltrated with LR White resins. The tissues were polymerized under ultraviolet light at -8°C for 3 days. Semi-thin (1 mm) sections were prepared to select areas for ultrathin (100 nm) sections. Sections were stained with ED-1 (1:100), IL-1β (1:25), TNF-α (1: 25) and TGF-β2 (1:25) antibodies followed by appropriate gold-conjugated secondary antibodies (respective, 1:100 diluted) and examined in an electron microscope at 60 kV. Immuno-electron microscopy controls included omission of primary antibody or staining of lung sections with anti-von Willebrand Factor antibody.

### Enzyme-linked immunosorbent linked assay

Lung samples were homogenized in Hank's balanced salt solution (HBSS) containing protease inhibitor cocktail (100 μl/10 ml; Sigma-Aldrich Co, MO, USA) in a ratio of 0.1 g of tissue in 1 ml of the solution, and centrifuged at 25,000 rpm at 4°C to collect the supernatant which was stored at -70°C in 100 μl aliquots. The ELISA kits for rat IL-1β, TNF-α and TGF-β2 were purchased from R & D systems, Inc., USA. Microtiter plates (Immulon 4 HBX, VWR CAN LAB, Canada) were coated with 100 μL of capture antibody and incubated overnight at room temperature. Non-specific bindings were blocked with 200 μL of 1% BSA. Then, standards and the samples in 100 μL quantities were incubated for 2 hours at room temperature. This was followed by incubation with 100 μL of biotinylated detecting antibodies for 2 hours at room temperature. Subsequently, plates were incubated with 100 μL of avidin-HRP (Vector laboratories, Inc., USA) for 40 minutes at room temperature. Finally, 100 μL of 3,3', 5,5'-tetramethyl-benzidine dihydrochloride (TMB) substrate (Mandel Scientific, ON, Canada) was added and incubated for 10–15 minutes at room temperature. After adequate color development, the reaction was stopped with 50 μl of 1 M sulfuric acid. In between each step until adding the substrate, plates were washed with PBS containing 0.05%-Tween20 (PBST). The optical densities were measured at 450 nm. Cytokine concentrations of test samples were determined using linear regression of standard curve and expressed as picograms per milliliter. The optimal concentrations for capture (TNF-α: 1 μg/ml, IL-1β:1 μg/ml and TGF-β2: 2 μg/ml) and detecting antibody (TNF-α: 1 μg/ml, IL-1β: 300 ng/ml and TGF-β2: 50 ng/ml) pair for each cytokine were titrated prior to running ELISA with test samples.

### Statistical Analysis

All values were presented as mean ± standard error (SE). We performed one-way ANOVA to compare granulocyte and ED-1 positive macrophage numbers at various time points following exposure to barn air or ambient air (control). Using two-way ANOVA, we examined the effect of exposure (barn or ambient air), effect of secondary challenge (saline or LPS) and the interaction effect between exposure and secondary challenge. ANOVA was followed by Tukey's *post-hoc *test (SigmaStat^®^, version 2.0 for Windows^® ^95, NT and 3.1, 1997; Chicago, IL, USA). Statistical significance was accepted at P < 0.05.

## Results

### BAL analyses

There were no differences in total and differential leukocyte counts among various groups in this study (P > 0.05; data not shown).

### Recruitment of granulocytes and PIMMs

**Figure 1:**
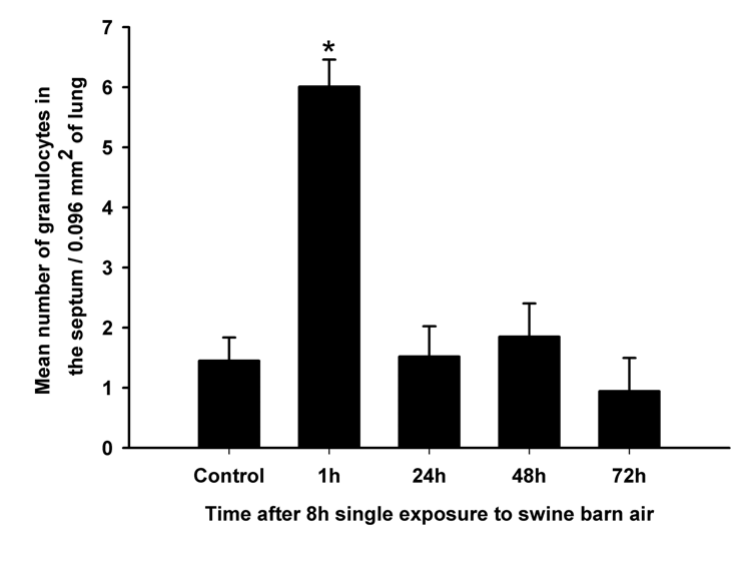
**Granulocytes**. Morphometric quantification of anti-granulocyte antibody stained granulocytes in the lung sections revealed an increase in granulocyte numbers at 1 hour post-exposure compared to the controls and other post-exposure time points (*, P < 0.05, Figure 1).

**Figure 2:**
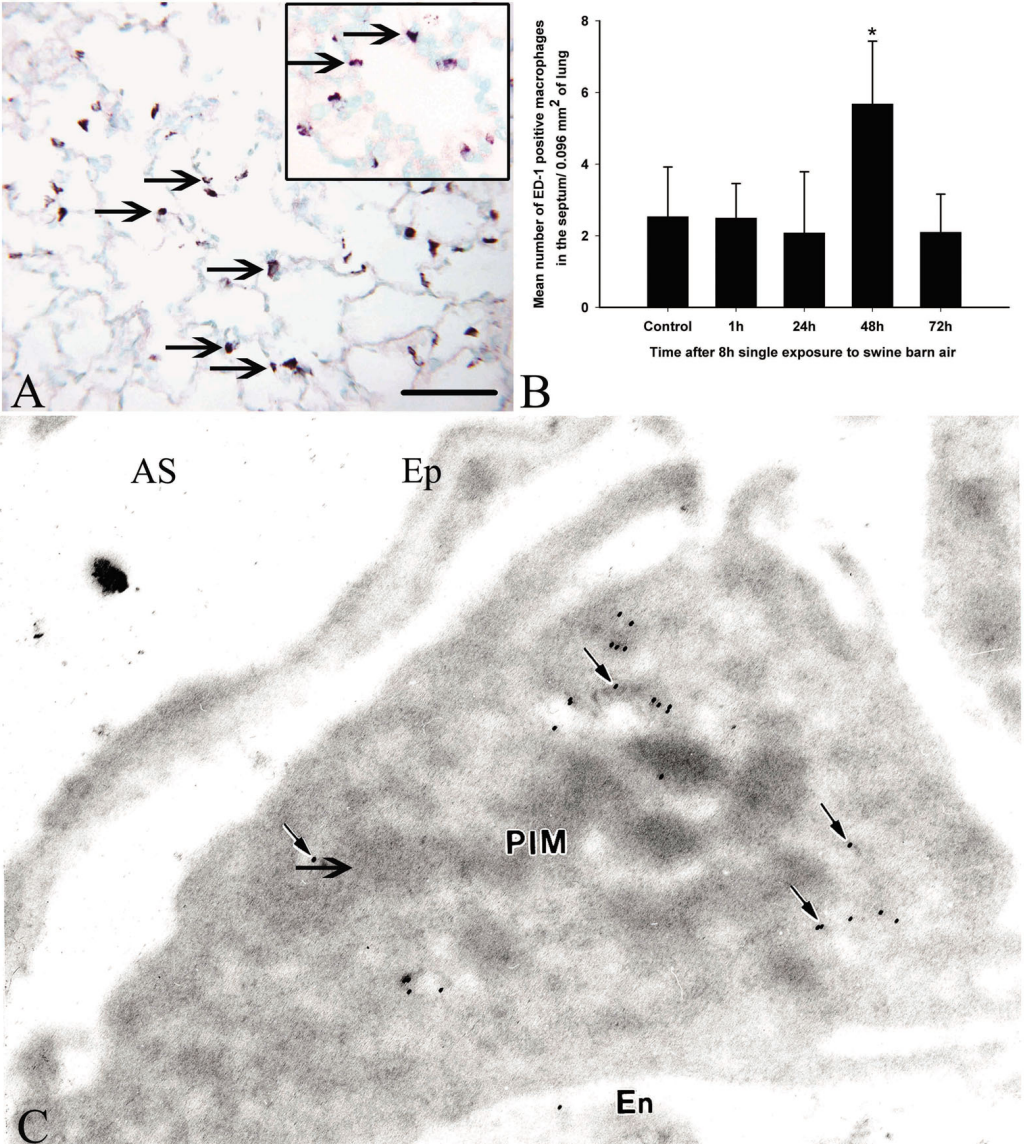
**Pulmonary intravascular monocytes/macrophages**. ED-1 antibody stained monocytes/macrophages in the lung septum (A, arrows and inset, bar = 50 μm) and morphometric quantification of septal cells positive for ED-1 antibody showed an increase in their numbers at 48 hours post-exposure compared to controls and other exposed groups (*, P < 0.05, Figure 2B). Pulmonary intravascular monocyte/macrophage (PIMM) shows gold particle (C, arrows) to indicate staining for ED-1 antibody (En- endothelium, Ep-epithelium and AS- alveolar space).

Numerical counts on lung sections stained with anti-granulocyte antibody showed an increase in granulocyte numbers in the septum at one hour after an 8 hour exposure compared to the controls and other post-exposure time points (P < 0.05, Figure 1). We used ED-1 antibody to stain rat monocytes/macrophages in the lung at both light and electron microscopic levels (Figure 2,A and 2C). The data showed an increase in ED-1 positive septal cells at 48 hours post-exposure compared to the controls and other exposed groups (P < 0.05, Figure 2B). The PIMM numbers returned to normal values by 96 hours and 120 hours post-exposure (data not shown). Immuno-electron microscopy confirmed ED-1 staining and intravascular location of PIMMs (Figure 2C).

### Response to secondary challenge

#### Histopathology

**Figure 3:**
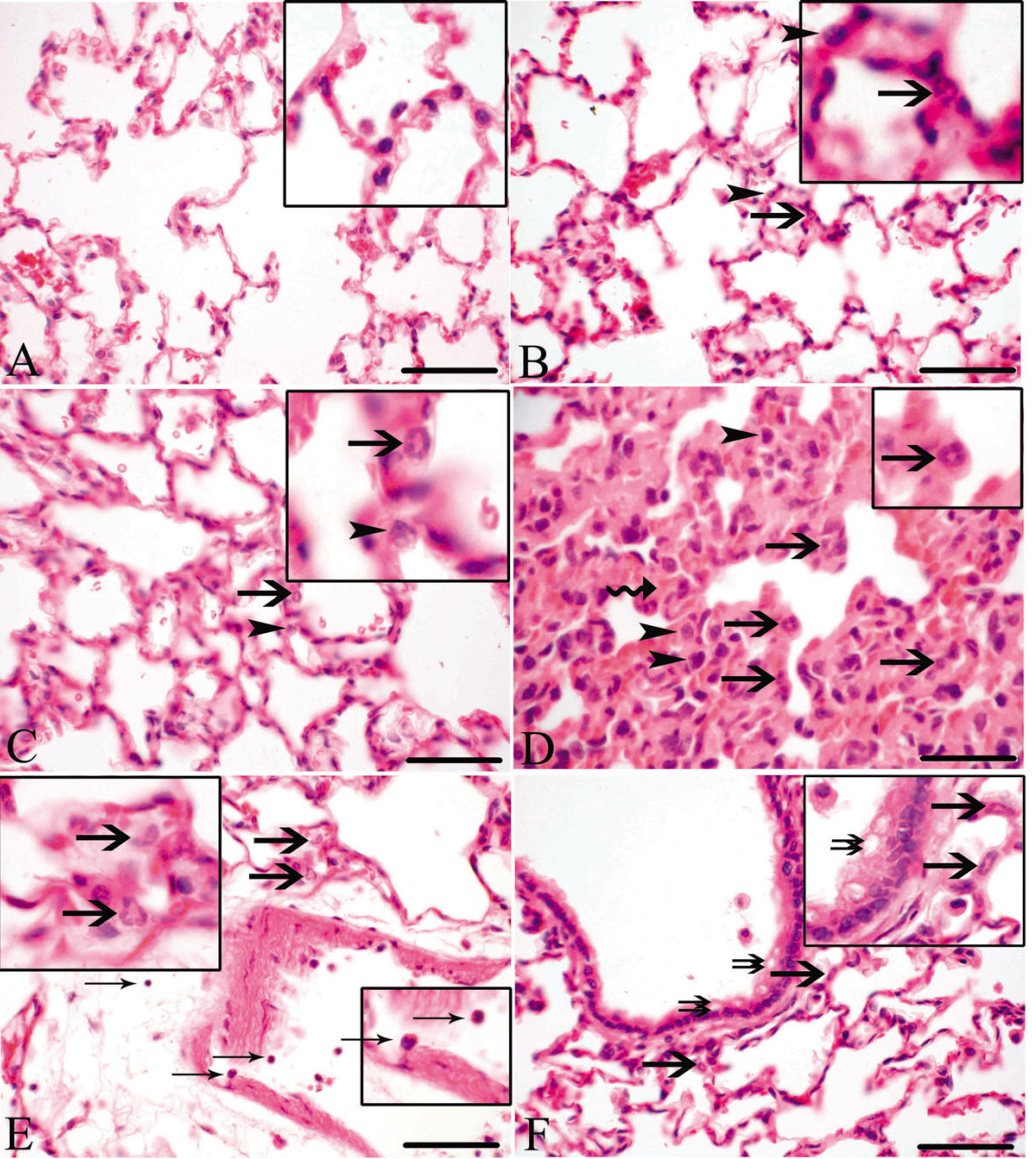
**Histopathology**. Lung sections from control rats showed normal histology (Figure 3A, inset) while lungs from rats exposed to barn and challenged with saline (Figure 3B and inset) and unexposed rats challenged with *E. coli *LPS (Figure 3C and inset) showed infiltration of neutrophils (arrows) and macrophages (arrowheads) into the lung septum. Lung sections from rats exposed to barn and challenged with *E. coli *LPS showed septal infiltration of neutrophils (D-F and insets; arrows), macrophages (D, arrow heads) and thickened septa (D, curved arrow), margination and attachment of leukocytes to the blood vessel wall along with perivascular infiltration (Figure 3E, thin arrows and inset) and damage to the bronchiolar epithelium (F, double arrows and inset). *Original magnification (A-F*): 400× and bar = 50 μm (A-F).

Lung sections from control rats showed normal histology of the septa and the alveolar spaces (Figure 3A). Rats exposed to barn and challenged with saline (Figure 3B) and unexposed rats challenged with *E. coli *LPS (Figure 3C) had infiltration of neutrophils and macrophages into the lung septum. Lung sections from rats exposed to barn air and challenged with *E. coli *LPS showed more infiltration of neutrophils and macrophages into the lung septum along with thickening of septa (Figure 3D), margination and sticking of leukocytes to the blood vessel wall, perivascular infiltration of inflammatory cells (Figure 3E) and damage to the bronchiolar epithelium (Figure 3F). Therefore, compared to other groups, rats exposed to the barn and challenged with *E. coli *LPS appeared to have more lung inflammation.

#### Increase in granulocytes following secondary challenge

**Figure 4:**
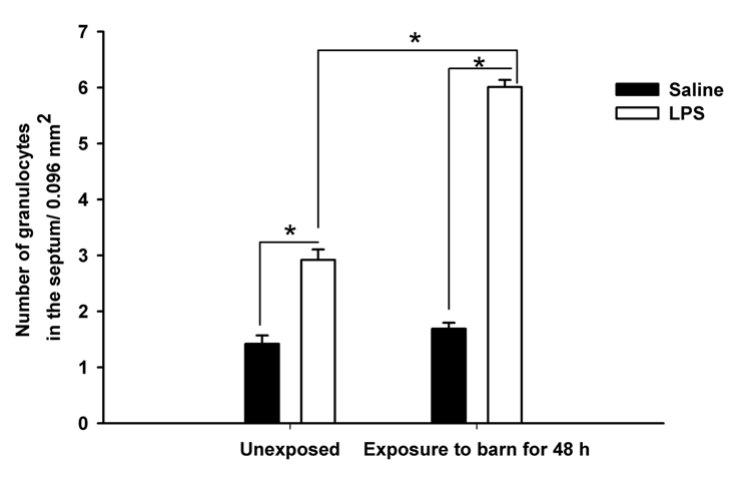
**Increased granulocyte numbers following a secondary challenge**. Rats exposed to the barn and challenged with LPS showed more number of granulocytes in the lung septum compared to barn exposed and saline challenged and LPS challenged unexposed rats. Within unexposed rats, LPS challenged rats showed more granulocytes compared to saline challenged ones (*, P < 0.001).

Numerical quantification of granulocytes in lung sections showed an effect of exposure, an effect of secondary challenge and an interaction between exposure and secondary challenge (P < 0.001 for all three, Figure 4). Within unexposed or barn exposed rats, those challenged with LPS contained more granulocytes in their lungs compared to those administered saline (P < 0.001). Rats exposed to the barn and challenged with LPS showed more granulocytes in the lung septum compared to unexposed LPS-treated rats (P < 0.001).

#### Expression and quantification of IL-1β

**Figure 5:**
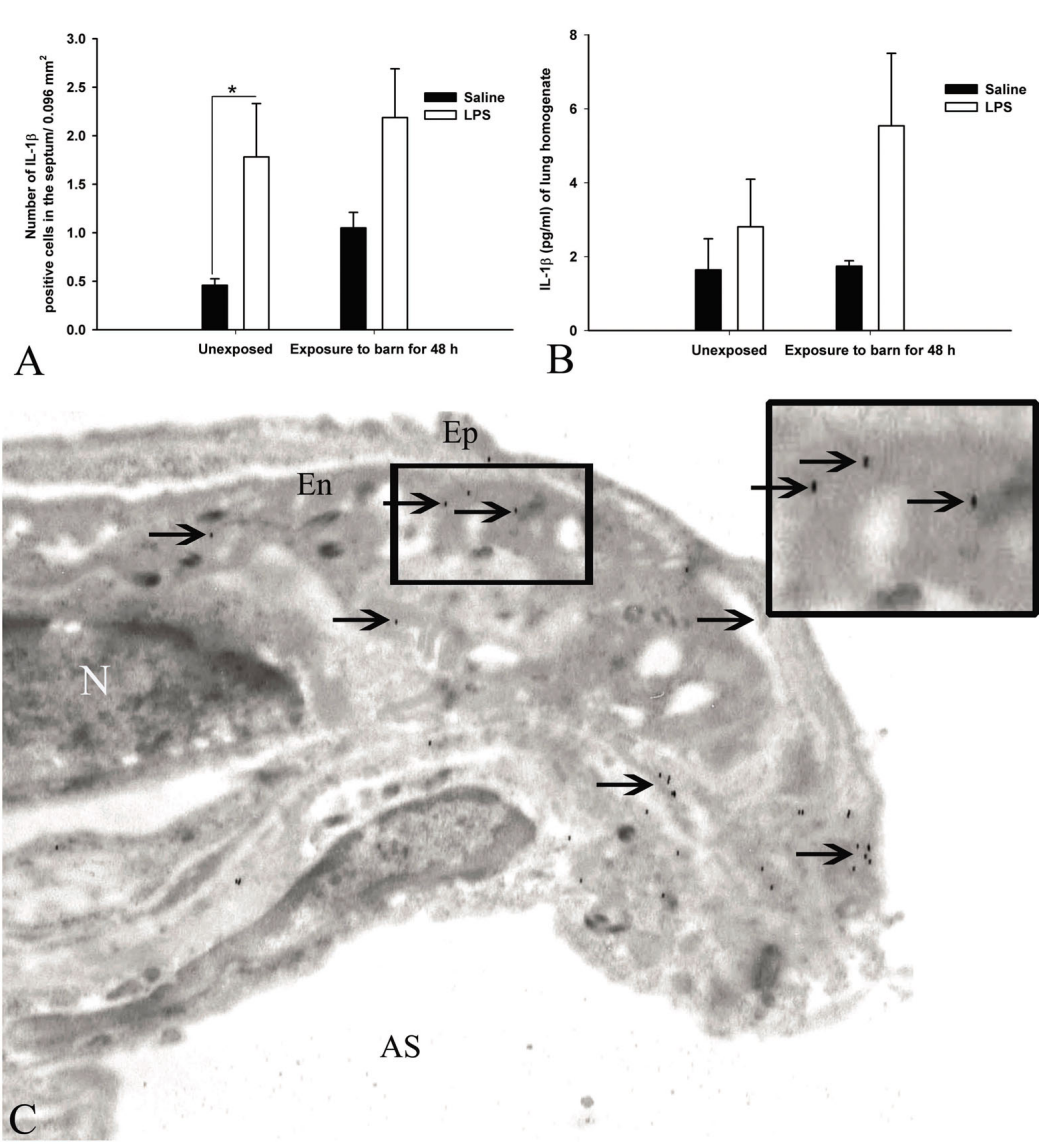
**Expression and quantification of IL-1β**: Quantification of cells stained with an anti-IL-1β antibody showed that unexposed rats treated with *E. coli *LPS contained more positive cells compared to saline treated unexposed (*, P < 0.026, Figure 5A). ELISA revealed no group differences in the concentrations of IL-1β (Figure 5B, P > 0.05). Immuno-electron microscopy using the same antibody localized IL-1β in pulmonary intravascular monocytes/macrophages (Figure 5C, arrows and inset).

Lung sections from unexposed rats treated with *E. coli *LPS contained more cells stained with IL-1β antibody compared to unexposed rats treated with saline (P = 0.026, Figure 5A) while none of the other groups differed significantly. ELISA showed no difference in IL-1β levels among the four groups (P > 0.05, Figure 5B). Immuno-electron microscopy localized IL-1β in PIMMs and the alveolar septum (Figure 5C, arrows and inset).

#### Expression and quantification of TNF-α

**Figure 6:**
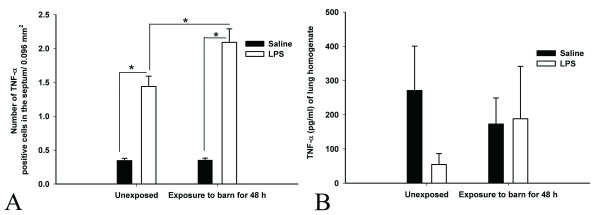
**Expression and quantification of TNF-α**: Quantification of cells stained with anti-TNF-α antibody showed that following *E. coli *LPS challenge, both unexposed and exposed groups contain more number of positive cells compared to saline treated both exposed and unexposed groups (*, P < 0.05, Figure 6A). Within LPS challenged groups, barn exposed rats contained more cells positive for TNF-α compared to unexposed rats (*, P = 0.005). ELISA on lung homogenates showed no differences in the concentrations of TNF-α among the groups (P > 0.05, Figure 6B).

Quantification of TNF-α positive cells in the septum showed an effect of barn air exposure (P = 0.041), an effect of secondary challenge (P < 0.001) and an interaction effect between barn air and secondary challenge (P = 0.046). Lungs from unexposed or barn-exposed rats challenged with *E. coli *LPS showed more number of cells positive for TNF-α compared to the respective saline-treated groups (P < 0.001, Figure 6A). Interestingly, rats exposed to the barn air and challenged with the *E. coli *LPS had more septal cells positive for TNF-α compared to the unexposed LPS-treated rats (P = 0.005, Figure 6A). ELISA on lung homogenates showed no differences in the concentrations of TNF-α among the four groups (P > 0.05, Figure 6B). Lung sections stained with TNF-α antibody demonstrated positive cells in the septa of barn-exposed rats and the cytokine was localized in PIMMs with immuno-gold electron microscopy (data not shown).

#### Expression and quantification of TGF-β2

**Figure 7:**
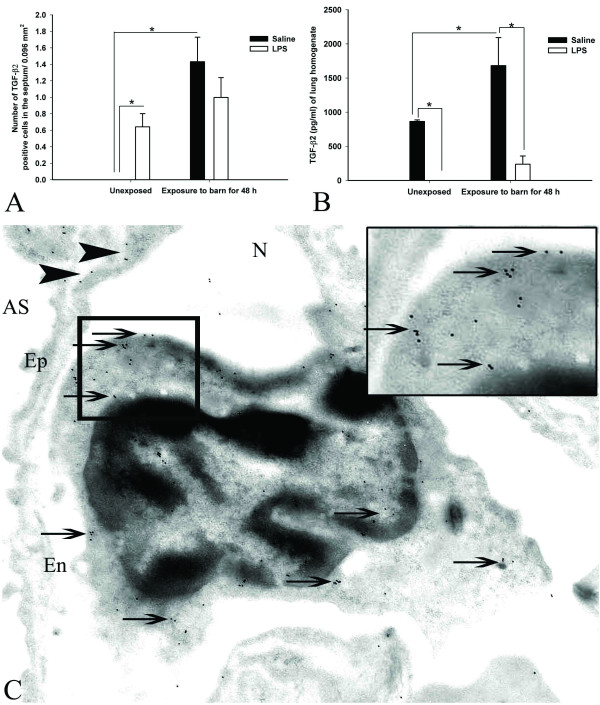
**Expression and quantification of TGF-β2**: Quantification of cells stained with TGF-β2 antibody showed increased numbers of positive cells in rats exposed to barn and challenged with saline at 48 hours post-exposure compared to saline treated unexposed controls (*, P < 0.05, Figure 7A). Within unexposed rats, LPS challenged rats contained more cells positive for TGF-β2 compared to saline challenged ones (*, P = 0.044, Figure 7A). ELISA showed higher concentration of TGF-β2 in the lung homogenates of saline-treated exposed or unexposed rats compared to the *E. coli *LPS-treated exposed or unexposed rats (*, P < 0.05, Figure 7B). Immuno-gold electron microscopy localized TGF-β2 in the cytoplasm and nucleus of pulmonary intravascular monocytes/macrophages (Figure 7C, arrows and inset) as well as in the lung endothelium and epithelium (Figure 7C, arrowheads).

Numerical counts of cells positive for TGF-β2 revealed an effect of exposure (P < 0.001, Figure 7A) and an interaction between exposure and secondary challenge (P = 0.020). Compared to unexposed rats treated with saline, unexposed LPS-challenged (P = 0.044) and exposed saline-treated rats (P < 0.0001) showed increased numbers of TGF-β2 positive cells. Quantification of TGF-β2 using ELISA showed an exposure effect (P = 0.039) and an effect of secondary challenge (P < 0.001). Among the unexposed rats, saline challenged rats showed higher concentrations of TGF-β2 compared to LPS challenged animals (P = 0.022, Figure 7B). Among the saline challenged rats, barn exposed rats showed higher levels of TGF-β2 compared to unexposed ones (P = 0.027, Figure 7B). Among the barn exposed rats, those given saline contained higher concentrations of TGF-β2 compared to the ones treated with LPS (P = 0.002, Figure 7B). Immuno-gold electron microscopy showed TGF-β2 staining in PIMMs, alveolar epithelium and capillary endothelium (Figure 7C).

## Discussion

The data reported in this paper show that a single exposure to the barn air induces acute lung inflammation including recruitment of granulocytes and PIMMs. The data further show that exposure to the barn air increases susceptibility for increased lung inflammation following a secondary challenge, which may be partially due to recruited PIMMs.

Although it has been known for some time that swine barn workers experience acute lung dysfunction including reduction in FEV1 and inflammation across a single shift in the barn [26-28], there has been a lack of reliable animal models to study cellular and molecular changes upon exposure to the barn air. Recently, we reported characterization of a rat model to investigate the pulmonary impact of exposure to the barn air [17]. Now, we have used this model to examine lung inflammation at various times points following a single 8 hour exposure to the barn air. It is well established that pig barn air contains significant amounts of endotoxin, dust and gases such as ammonia [29,30]. The data showed significant increases in granulocyte numbers in the lung septum and the bronchiolar walls (Data not shown) at one hour following the 8 hour exposure to barn air. Recruitment of granulocytes in lungs of barn exposed rats is consistent with the recently reported production of IL-8, a potent chemoattractant for granulocytes, by airway epithelial cells exposed to the barn dust in vitro [31]. Furthermore, BAL fluid collected from human workers following a single 8 hour shift contains higher levels of IL-8 [11]. Therefore, expression of chemoattractants such as IL-8 following exposure to the barn air may be instrumental in provoking recruitment of granulocytes and induction of acute lung inflammation following a single exposure to pig barn air.

Neutrophil migration is typically followed by recruitment of monocytes/macrophages. We observed a novel increase in ED-1 positive monocytes/macrophages cells in the alveolar septa at 48 hours after barn exposure compared to other time points. ED-1 antibody recognizes a lysosomal protein and has previously been used to recognize rat monocytes/macrophages [32-34]. Because of resolution limits of light microscopy, we used immuno-electron microscopy to confirm the intravascular location of ED-1 positive monocytes/macrophages. The recruitment pattern of PIMMs in barn-exposed rats determined through ED-1 staining is similar to that observed following a single bacterial challenge [19,20]. However, transient PIMM recruitment induced by barn air is different from more permanent PIMM accumulation observed following bile duct ligation [35]. Because we did not observe any changes in BAL neutrophil and monocyte/macrophage cell counts, it appears that exposure to barn air predominantly induced vascular accumulation of neutrophils and PIMMs. It is also possible that a strong enough chemotactic gradient was not induced following a single exposure to barn air. Nevertheless, the data show a single cycle of acute lung inflammation is induced following an 8 hour exposure pig barn air.

We also examined the response of barn exposed rats specifically in the context of recruited PIMMs to a secondary challenge. This was necessitated because resident pulmonary intravascular macrophages in cattle, sheep and horses are credited with induction of robust lung inflammation [36,22,37]. Furthermore, we have recently reported that PIMMs recruited following an intraperitoneal injection of *E. coli *bacteria alter lung susceptibility to a secondary challenge with *E. coli *LPS [20]. Our data show that LPS treatment of barn-exposed rats compared to normal rats resulted in a higher accumulation of granulocytes and increased number of cells positive for TNF-α but not IL-1β in the lungs. Interestingly, ELISA did not reveal differences among any of the groups for concentration of IL-1β and TNF-α in lung tissues. We do not know the reasons for the discrepancy between histologic and ELISA results for TNF-α. Differences in sensitivities of the two methods could be a contributing factor. Immuno-histochemistry may have detected the residual intracellular cytokines while most of the cytokines were secreted into the circulation thus resulting in lack of differences between groups with ELISA. We still believe that ELISA is a more powerful and sensitive method for molecular quantification. It is possible that we may have missed the window of increased concentrations of the assayed cytokines in rat lungs. Nevertheless, it is important to note that granulocyte numbers were higher in LPS-treated rats that contained PIMMs. Because granulocyte migration requires vascular expression of cytokines and adhesion molecules and is based on the localization of IL-1β and TNF-α in PIMMs, we believe that recruited PIMMs may have played a major role in provoking increased migration of granulocytes into inflamed lungs.

Inflammation is manifested through a complex interplay of inflammatory and pro-inflammatory cytokines. Therefore, we also examined the expression of TGF-β2, which is classified as an anti-inflammatory cytokine involved in tissue repair and remodeling [38-42]. We noticed highest lung expression of TGF-β2 in conjunction with peak recruitment of PIMMs at 48 hours after exposure to the barn. Interestingly, rats treated with LPS at 48 hours post-exposure or without barn exposure showed reduced expression of TGF-β2. These data suggest that TGF-β2 may play anti-inflammatory roles in lung inflammation induced following exposure to the barn air, and that its expression may be suppressed to manifest acute inflammation engendered through LPS treatment of the exposed rats. PIMMs showed TGF-β2 in addition to IL-1β and TNF-α to underscore the complex and multifaceted roles of monocytes/macrophages in lung inflammation. It appears that the relative balance of cytokines produced by monocytes/macrophages results in fine and tight regulation of inflammatory processes.

## Conclusion

We report novel recruitment of PIMMs in barn-exposed rats and increased lung inflammation in exposed rats subjected to a secondary challenge with LPS. It appears that recruited PIMMs may be involved in increased inflammation through their contribution of multiple cytokines.

## Competing interests

The author(s) declare that they have no competing interests.

## Authors' contributions

LNAG carried out the experiment, immunohistochemistry, ELISA, statistical analyses and drafted the manuscript. TLS helped during the experiment, CC did the image analyses of histological sections and took pictures, prepared figures and helped in manuscript preparation. BS conceived of the study, participated in its design, performed immuno-electron microscopy and participated in the preparation of the manuscript. All authors have read and approved the final manuscript.
